# Decreased ANGPTL4 impairs endometrial angiogenesis during peri‐implantation period in patients with recurrent implantation failure

**DOI:** 10.1111/jcmm.15696

**Published:** 2020-08-03

**Authors:** Mingyang Li, Jingwen Hu, Lihua Yao, Minzhi Gao

**Affiliations:** ^1^ Center for Reproductive Medicine Ren Ji Hospital School of Medicine Shanghai Jiao Tong University Shanghai China; ^2^ Shanghai Key Laboratory for Assisted Reproduction and Reproductive Genetics Shanghai China

**Keywords:** angiogenesis, ANGPTL4, endometrial receptivity, recurrent implantation failure, rosiglitazone

## Abstract

Insufficient endometrial angiogenesis during peri‐implantation impairs endometrial receptivity (ER), which contributes to recurrent implantation failure (RIF) during in vitro fertilization and embryo transfer (IVF‐ET). Angiopoietin‐like protein 4 (ANGPTL4) acts as a multifunctional secretory protein and is involved in the regulation of lipid metabolism and angiogenesis in various tissues including the endometrium. Herein, we found decreased ANGPTL4 expression in endometrial tissue and serum during peri‐implantation period in 18 RIF‐affected women with elevated uterine arterial impedance (UAI) compared with the pregnancy controls. ANGPTL4 and peroxisome proliferator‐activated receptor gamma (PPARγ) expression were up‐regulated upon decidualization on human endometrial stromal cells (HESCs). Rosiglitazone promoted the expression of ANGPTL4 in HESCs and human umbilical vein endothelial cells (HUVECs) via PPARγ. ANGPTL4 promoted the proliferation, migration and angiogenesis of HUVECs in vitro. Our results suggest that decreased abundance of ANGPTL4 in endometrial tissues impairs the endometrial receptivity via restraining endometrial angiogenesis during decidualization; while rosiglitazone‐induced ANGPTL4 up‐regulation in hESCs and HUVECs through PPARγ. Therefore, ANGPTL4 could be a potential therapeutic approach for some RIF‐affected women with elevated UAI.

## 1 INTRODUCTION

Embryo implantation is a key process for continuous pregnancy. About 1 in 5 pregnancies end in an early pregnancy loss, with aneuploidy being a significant single factor of early pregnancy failure, while about 50% of early pregnancy loss is due to implantation failure.^1^ In addition, implantation failure remains a major limiting factor for assisted reproductive technology (ART). The continuous pregnancy rate is 20–30% in the therapy of in vitro fertilization and embryo transfer (IVF‐ET) relatively, possibly less effective than normal human pregnancy.^2^ In spite of the application of pre‐implantation genetic diagnosis (PGD) and the transference of a single euploid blastocyst of good quality, the cumulative pregnancy rate after three in vitro fertilization (IVF) cycles has only improved to 40–55%.^3^ Thus, recurrent implantation failure (RIF) due to impaired endometrial receptivity still remains a challenging and frustrating problem in IVF.^4^ There are no strict criteria defined for RIF yet, but general consensus refers to a failure to achieve clinical pregnancy after transferring no less than four embryos of good quality in at least three fresh or frozen cycles in women under the age of 40.^5^, ^6^, ^7^

Impaired endometrial receptivity (ER) is considered to be the leading cause of implantation failure in IVF.^8^ The synchronization of decidualization and vascular processes during the window of implantation (WOI) period is essential for the development of a receptive endometrial environment for embryo implantation.^9^ Decidualization of human endometrial stromal cells (hESCs) involves obvious morphological and functional differentiation, which includes edema of endometrial stromal, proliferation and differentiation of stromal cells, leucocyte invasion, vascular remodelling, enhancement of vascular permeability and angiogenesis.^10^ Insufficient endometrial angiogenesis during WOI inevitably affect the establishment of appropriate endometrial receptivity, leading to embryo implantation failure.^11^ Angiopoietin‐like protein 4 (ANGPTL4) functions as a multifunctional secretory protein and is involved in the regulation of lipid metabolism, glucose homoeostasis, inflammation, angiogenesis and vascular permeability .^12^ The transcriptional regulation of ANGPTL4 can be modulated by a number of factors including glucocorticoids, free fatty acids, peroxisome proliferator‐activated receptors (PPARs) agonists, transforming growth factor‐β (TGF‐β) and hypoxia inducible factor‐1α (HIF‐1α).^12^, ^13^, ^14^ Moreover, it has been reported that the activation of PPARγ promotes the expression of ANGPTL4 in trophoblast, thereby enhances placental angiogenesis.^15^ However, the role of ANGPTL4 in endometrial angiogenesis during decidualization has not been reported yet.

Herein, we have detected decreasing ANGPTL4 abundance in serum and endometrial tissues of RIF‐affected women with elevated uterine arterial impedance (UAI) during peri‐implantation period. Moreover, we explored the effect of rosiglitazone (a PPARγ agonist) on the expression of ANGPTL4. The detailed functions of ANGPTL4 involved in decidualization were further conducted in immortal hESCs and human umbilical vein endothelial cells (HUVECs) cell lines.

## 2 MATERIALS AND METHODS

### 2.1 Experimental subjects

All patients in the study were recruited from the Center for Reproductive Medicine, Ren Ji Hospital, School of Medicine, Shanghai Jiao Tong University between March 2016 and April 2018, who underwent IVF or intracytoplasmic sperm injection (ICSI) treatment due to tubal and/ or male factor. The infertile patients were routinely monitored for endometrial thickness and uterine artery blood flow by transvaginal sonography during peri‐implantation period. The values of uterine artery peak systolic velocity/ end diastolic velocity (S/D) over 6 on both sides were defined as elevated UAI. We retrospectively recruited 18 RIF patients with elevated UAI, and 18 control patients who achieved clinical pregnancy the transference cycle following endometrial biopsy collection. Women reporting conditions such as polycystic ovarian syndrome, endometriosis, endometrial polyps and uterine fibroids, uterine malformations, history of ovarian surgery, history of pelvic tuberculosis, thyroid dysfunction, hyperprolactinemia, thrombotic disorders and immune infertility were excluded. All women had normal body mass index (BMI; 18‐24 kg/m^2^), regular menstrual cycle, normal basal serum follicle stimulating hormone (FSH) level on the third day of the last menstruation (FSH < 10 IU/L) and no hormone therapy and aspirin intake within 3 months before admission. The clinical features of recruited patients are displayed in Table 1. The study was verified by Ren Ji Hospital ethics committee (No.2017041409). Informed consent forms were obtained from all patients.

**Table 1:** Comparison of general clinical characteristics between the control and RIF groups

Clinical features	Control (n = 18)	RIF (n = 18)
Age (year)	28.83 ± 4.62	30.00 ± 3.29
Infertility duration (year)	2.72 ± 1.87	4.44 ± 3.26
BMI (kg/m^2^)	21.17 ± 2.62	21.77 ± 4.38
AMH (ng/mL)	5.09 ± 2.15	5.20 ± 2.64
basal FSH (IU/L)	6.69 ± 1.41	6.62 ± 2.48
basal LH (IU/L)	5.00 ± 1.70	4.45 ± 1.86
basal E_2_ (pg/mL)	35.02 ± 21.58	36.89 ± 17.54
AFC	14.00 ± 4.42	16.56 ± 5.70
EM thickness (mm)	9.04 ± 1.33	8.97 ± 1.58
PI	2.05 ± 0.47	2.36 ± 0.34*
RI	0.81 ± 0.05	0.84 ± 0.02**
S/D	5.72 ± 1.38	6.56 ± 1.05*

Note

Abbreviation: AFC, antral follicle counting; EM thickness, endometrial thickness; PI, uterine arterial pulsatility index; RI, uterine arterial resistance index; S/D, uterine artery systolic peak flow rate/end diastolic flow rate.

The data are shown as the mean ± SD values. *P < .05, vs. control; ** *P* < .01, vs. control.

### 2.2 Collection of endometrial tissues and blood samples

Peripheral blood and endometrial biopsies were obtained from 36 recruited patients on Day LH + 7. The follicle growth was traced by ultrasound monitoring (Prosound, Aloka, Japan) and urine luteinizing hormone (LH) detection in a natural cycle. The day on which a dominant follicle reached 18 mm, together with positive urinary LH was defined to be the day of LH 0; the window phase for implantation was defined as the day of LH + 7. Serum was taken after centrifugation of peripheral blood to determine the concentrations of ANGPTL4 in serum. Endometrial samples were collected using an endometrial catheter (LILYCLEANER, Shanghai Jiabao medical healthcare science and technology Ltd. China). All tissues were washed twice with physiological saline, then 1/3 was fixed in paraformaldehyde for 24 h and embedded in paraffin, and the rest was frozen in liquid nitrogen and stored at −80°C for RNA and protein extraction.

### 2.3 Immunohistochemistry

Localization of PPARγ and ANGPTL4 in endometrial tissues was performed by immunohistochemistry (IHC). In brief, the fixed endometrial paraffin sections obtained from the RIF and control groups were cut in 4μm section and treated with dimethylbenzene and alcohol. Then the sections incubated in citric acid antigen retrieval buffer were heated in a microwave oven. Subsequently, the slides were washed in phosphate buffered saline (PBS), blocked in 3% H_2_O_2_ for 30 min, blocked in 3% BSA for 1 h at room temperature, incubated with primary antibodies (rabbit anti‐ANGPTL4, 1:200, Proteintech; rabbit anti‐PPARγ, 1:200, Proteintech) overnight at 4°C and incubated with goat anti‐rabbit secondary antibody (RTU vectorstain, Vector, USA) for 1 h at room temperature. Finally, the sections were observed under light microscope (Zeiss, Jena, Germany).

### 2.4 Cell culture, in vitro decidualization and treatments

The immortalized HESC line, a kindly gift from Professor Haibin Wang of Xiamen University in China, has underwent the process of immortalization by transfection of telomerase (hTERT) using a retroviral system using pA317 hTERT‐expressing cell line and is karyotypically, morphologically and phenotypically similar to the primary parent cells.^16^ Human umbilical vein endothelial cells (HUVECs) are gifted from Chinese Academy of Science Cell Bank. Both cell lines were cultured in Dulbecco modified eagle medium DMEM/F‐12 (Gibco, USA), supplemented with 10% carbon‐stripped foetal bovine serum (Biological Industries, Israel) and 5 μg/L antibiotics (Gibco). HESCs were incubated with DMEM/F12 supplemented with 2% charcoal‐stripped FBS, N^6^,2’‐O‐Dibutyryladenosine 3’,5’‐cyclic monophosphate sodium salt (Dibutyryl cAMP; final concentration of 0.5 mmol/L; Sigma‐Aldrich) and progesterone (P_4_; final concentration of 1 μmol/L; Sigma‐Aldrich) for 4 days to induce decidualization in vitro, and the medium was replaced every other day. On the 4th day, culture medium was recovered for enzyme‐linked immunosorbent assay (ELISA), and the extent for decidualization was also checked by studying the change of morphology of decidual cells.

### 2.5 Real‐time PCR

According to the manufacturer's instructions, total RNA was extracted by a total RNA isolation kit (FOREGENE, China) and stored at – 80°C until analysis. A total of 1000 ng RNA was used for cDNA synthesis by a PrimeScript RT Master Mix Perfect Real Time kit (Toyobo, Japan). Real‐time PCR was performed in ABI Prism7300 machine (Applied Biosystems, USA) using the SYBR green master mix (Toyobo, Japan). The sequences of the primers designed by Primer Premier Software and obtained from Shanghai Sangon Biotech Co. Ltd are as follows:

PPARγ (forward): 5’‐CCTCATGGCAATTGAATGTCG‐3’.

(reverse): 5’‐CCGGAAGAAACCCTTGCATC‐3’.

ANGPTL4 (forward): 5_‐GGCTCAGTGGACTTCAACCG‐3_.

(reverse): 5’‐CCGTGATGCTATGCACCTTCT −3’.

Insulin‐like growth factor binding protein 1 (IGFBP1) (forward): 5’‐GGCACAGGAGACATCAGGAGAA‐3’.

(reverse): 5’‐GGTAGACGCACCAGCAGAGT‐3’.

Prolactin (PRL) (forward): 5’‐CATATTGCGATCCTGGAATGAG‐3’.

(reverse): 5’‐GATGAACCTGGCTGACTATCA‐3’.

ACTB (forward): 5’‐GGGAAATCGTGCGTGACATTAAG‐3’.

(reverse): 5’‐TGTGTTGGCGTACAGGTCTTTG‐3’.

PCR was performed under the following conditions: 3 min at 95°C, 40 cycles of 15 s at 95°C, 20 s at 60°C and 20 s at 72°C and 5 min at 72°C. Melting curve analyses were checked to verify the identity of product. PCR was done in triplicate and results relative to ACTB were calculated and differences were quantified using the 2^‐⊿⊿Ct^ method.^17^

### 2.6 Western blot analysis

Total protein was extracted using RIPA buffer (Beyotime, Shanghai, China) with protease and phosphatase inhibitors (Roche, Penzberg, Germany). Each sample was loaded with 40 μg of protein on an SDS polyacrylamide gel for electrophoresis and subsequently transferred to polyvinylidene fluoride membranes. The membranes were blocked in 5% no‐fat milk for 60min at room temperature and then were incubated overnight at 4°C with primary antibodies as follows: rabbit anti‐ANGPTL4 (polyclonal, dilution 1:1000; Proteintech); rabbit anti‐PPARγ (polyclonal, dilution 1:1000; Proteintech); and mouse anti‐GAPDH (monoclonal, dilution 1:5000; Proteintech). Next day, membranes were incubated for 60 min at room temperature with a goat anti‐rabbit peroxidase‐conjugated secondary antibody (dilution 1:5000; Proteintech) or a goat anti‐mouse peroxidase‐conjugated secondary antibody (dilution 1:5000; Proteintech). An enhanced chemiluminescence detection system (Millipore) was used to visualize protein bands. The same blot was probed for GAPDH (dilution 1:5000; Proteintech) as an internal loading control. The bands were visualized using the G‐Box iChemi Chemiluminescence Image Capture System (Syngene, MD, USA).

### 2.7 Enzyme‐linked immunosorbent assay

According to the manufacturer's protocol, the concentrations of ANGPTL4 in the serum and culture medium were measured using an enzyme immunoassay kit (R&D systems, MN, USA) with a detecting range from 1.25 to 80 ng/mL. The serum and culture medium were performed a 2‐fold dilution before detection. Each sample was run in duplicate.

### 2.8 Small interfering RNA and transfection

Specific small interfering RNA (siRNA) was obtained from Biotend (Shanghai, China); Approximately 5 × 10^5^ cells per dish of hESCs were plated in the non‐phenol red medium without antibiotics and incubated for 12 h, at about 60% confluence, siRNA (final concentration of 25 nM) and lipofectamine 3000 (Invitrogen, 5μl/well) diluted in Opti‐MEM (Gibco) were transfected into the hESCs in six‐well plates. The medium was replaced 12 h later and cells were treated for in vitro decidualization for 4 days. The sequences of the target genes were as follows:

Human *ANGPTL4* siRNA, 5’‐GAAACUUGUGGACAGAGAAdTdT‐3’; Randomly scrambled siRNA, 5’‐UUCUCCGAACGUGUCACGUdTdT‐3’.

### 2.9 Cell proliferation assays in HUVECs

Cell proliferation ability was determined using the Cell Counting Kit‐8 (CCK‐8) assay (MedChemExpress, USA) according to the manufacturer's instructions. HUVECs transfected with small interfering RNA (final concentration of 25 nM) were seeded in a 96‐well plate at a density of approximately 1 × 10^4^ cells/well in 100μL of culture medium and incubated for 12 h at 37°C, followed by treatments with rosiglitazone (10 μM, Tocris Bioscience, United Kingdom) or recombinant human ANGPTL4 (100 nM, R&D systems, USA) for 0, 24, 48, 72 h respectively. HUVECs were incubated with DMEM/F12 supplemented with 10% charcoal‐stripped FBS. 10 μL of CCK‐8 solution was added to each well of the plate by a repeating pipettor. After being incubated for 3 h in the incubator, the absorbance of each well was read at 450 nm by a microplate reader (Infinite M200 PRO, Tecan, Switzerland).

### 2.10 Wound‐healing assays

HUVECs were incubated with DMEM/F12 supplemented with 10% charcoal‐stripped FBS in six‐well plates. After siRNA‐mediated knockdown of ANGPTL4, HUVECs were treated with 10 μM rosiglitazone or not. The control groups were treated with 10 μM rosiglitazone or 100 nM rhANGPTL4 after transfecting with scrambled siRNA. A wound was made using a sterilized 200 µL pipette tip. The scratch area between two sides of the wound was pictured and measured immediately (0 h) and again at 24 h and 48 h after wounding using the Image J 1.46r. Wound closure rates were calculated by subtracting the area of the wound at 24h or 48 h from that of the 0 h time point.

### 2.11 Tube formation assay

Tube formation assay was performed to observing angiogenesis as follows: growth factor reduced Matrigel (BD Biosciences, USA) was placed in a 48‐well cell culture plate (150 μL/well) and incubated at 37°C for 30 min. HUVECs transfected with small interfering RNA (final concentration of 25 nM) were seeded at approximately 2 × 10^4^ cells onto the Matrigel‐coated wells, followed by treatment with 10 μM rosiglitazone. Control groups were treated with either 10 μM rosiglitazone or 100 nM recombinant human ANGPTL4 and incubated at 37°C for 24 h. Tube formation was observed and imaged under an inverted microscope. Total tube length, numbers of branches and meshes were analysed by Image J 1.46r.

### 2.12 Statistical analysis

All data were analysed by SPSS statistical software (IBM 25.0, USA). The values are shown as the means ± SD. Differences among the groups were compared for significance using paired or unpaired student's t test or one‐way ANOVA. Correlation between the variables was performed using Pearson analysis. All experiments were repeated at least three times. Differences between groups were considered as statistically significant at *P* < .05.

## 3 RESULTS

### 3.1 Comparison of basal clinical characteristics between the two groups

By comparing basal clinical characteristics of two groups, there were no significant differences in average age, infertility duration, BMI, anti‐Mullerian hormone (AMH), basal FSH, basal LH, basal estradiol (E_2_), antral follicle count (AFC) and endometrial thickness between the RIF group and the control group (all *P* > .05). However, the three important uterine artery blood flow parameters, uterine arterial pulsatility index (PI), uterine arterial resistance index (RI) and uterine artery S/D, were significantly higher in the RIF group than those in the control group (*P* < .05) (Table 1).

### 3.2 Decreased abundance of ANGPTL4 in endometrial tissues and serum of RIF patients

**Figure 1:**
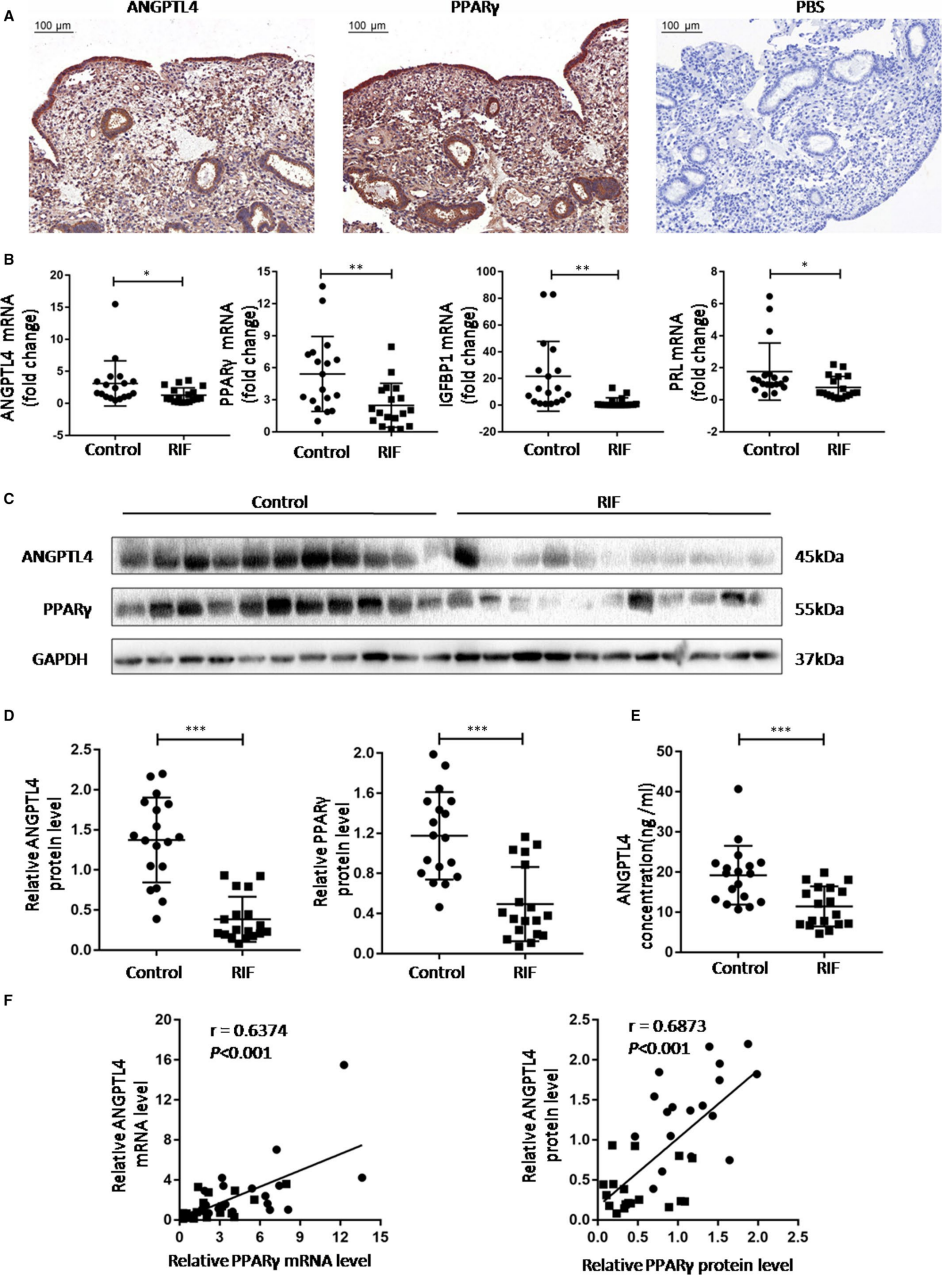
Expression of ANGPTL4 and PPARγ in endometrial tissues and serum of RIF patients and fertile women. (A) Immunohistochemical staining of ANGPTL4 and PPARγ in endometrium. (B) The mRNA levels of ANGPTL4, PPARγ, IGFBP1and PRL in endometrial tissues of RIF patients (n = 18) and fertile women (n = 18). (C and D) Protein levels of ANGPTL4 and PPARγ in endometrial tissues (n = 18 for each group). (E) Concentrations of ANGPTL4 in the serum from RIF patients (n = 18) and control group (n = 18). (F) Correlation analysis between the mRNA and protein levels of ANGPTL4 and PPARγ in endometrial tissues among all subjects (n = 36). Scale bar = 100 μm. The data are shown as the mean ± SD values. *P < .05, **P < .01 and ***P < .001 compared with corresponding control. Blots are representative

Immunochemical staining of endometrial tissue showed that both PPARγ and ANGPTL4 were present in endometrial cavity epithelium, glandular epithelium, vascular endothelium and endometrial stromal cells (Figure 1A). We found that the mRNA levels of ANGPTL4 and PPARγ in the RIF group (n = 18) in endometrial tissues were significantly lower than those in the control group (n = 18). Meanwhile, mRNA levels of endometrial decidualization biomarkers including IGFBP1and PRL were significantly reduced, indicating impaired decidualization in the RIF group (Figure 1B). In addition, protein levels of PPARγ and ANGPTL4 were significantly reduced in the RIF group compared with the control group (Figure 1C, D). Consistently, the concentrations of ANGPTL4 in the serum of the RIF group were significantly lower than those of the control group (Figure 1E). Furthermore, correlation analysis showed a positive correlation between the abundance of ANGPTL4 and PPARγ in all subjects (n = 36) (Figure 1F), indicating a functional interaction between PPARγ and ANGPTL4 in vivo.

### 3.3 PPARγ and ANGPTL4 both increased upon decidualization of hESCs in vitro

**Figure 2:**
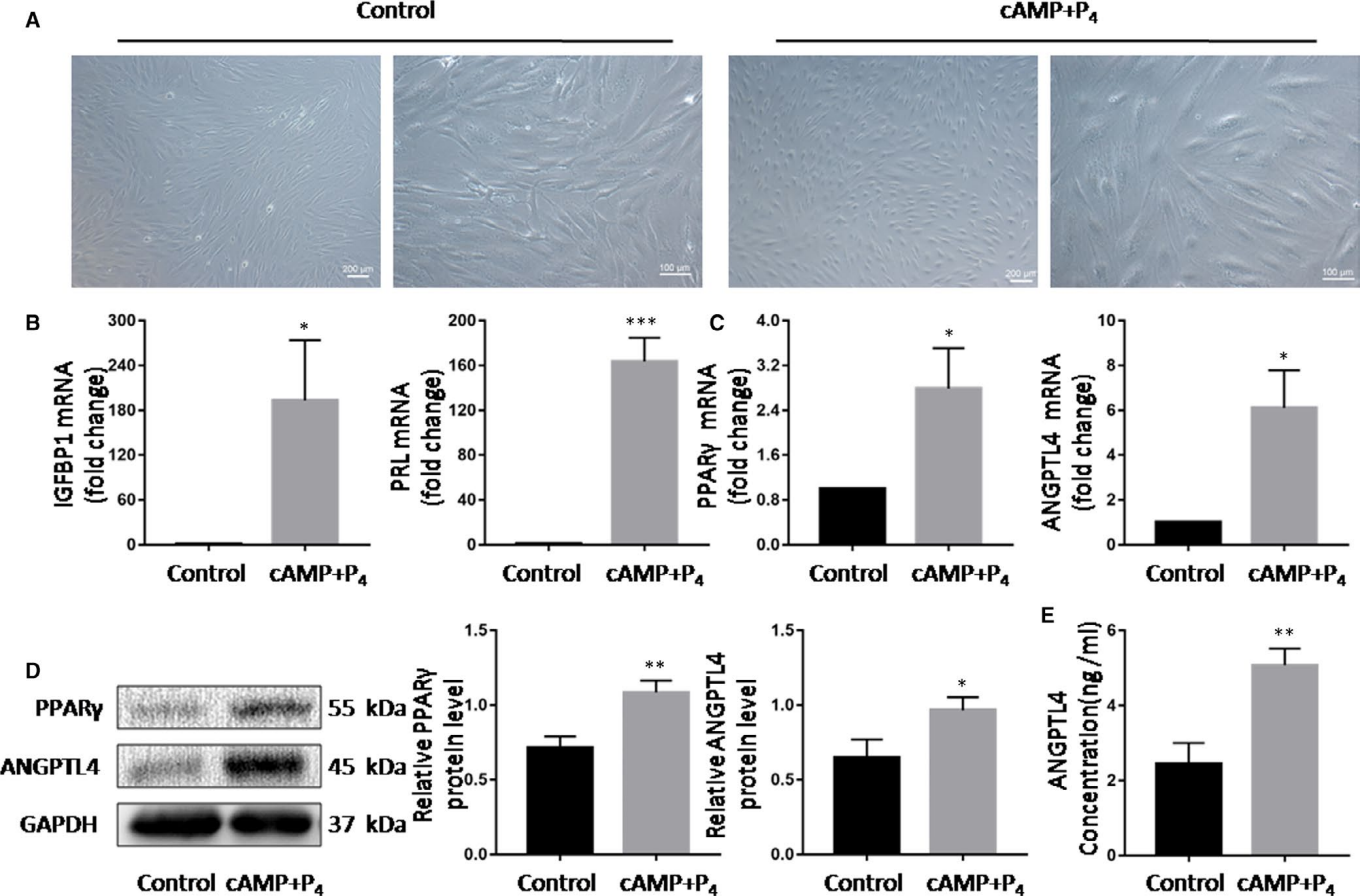
Changes of IGFBP1, PRL, PPARγ and ANGPTL4 in hESCs after decidualization. (A) Cell morphologies of hESCs on day 0 and 4 after in vitro decidualization. (B) The mRNA levels of IGFBP1, PRL, PPARγ and ANGPTL4 in hESCs after in vitro decidualization. (C) Protein levels of PPARγ and ANGPTL4 in hESCs after in vitro decidualization. (D) Concentrations of ANGPTL4 in culture medium of hESCs after in vitro decidualization. The data are shown as the mean ± SD values of at least three independent experiments performed. *P < .05 and **P < .01 compared with corresponding control

To analyse the expression of PPARγ and ANGPTL4 during decidualization, we used cAMP and P_4_ to induce decidualization on hESCs in vitro. These cells were larger and plumper than those untreated on day 4 (Figure 2A). Meanwhile, the mRNA levels of the decidualization markers IGFBP1 and PRL increased significantly, indicating that decidualization was successfully performed in vitro (Figure 2B). During decidualization, the abundance of PPARγ and ANGPTL4 mRNA was significantly up‐regulated compared to untreated cells (Figure 2C). Furthermore, western blotting demonstrated that both PPARγ and ANGPTL4 protein abundance increased after decidualization (Figure 2D). Meanwhile, increased ANGPTL4 in culture medium was detected by ELISA on the 4th day of decidualization (Figure 2E).

### 3.4 PPARγ mediates rosiglitazone‐induced up‐regulation of ANGPTL4 in hESCs and HUVECs

**Figure 3:**
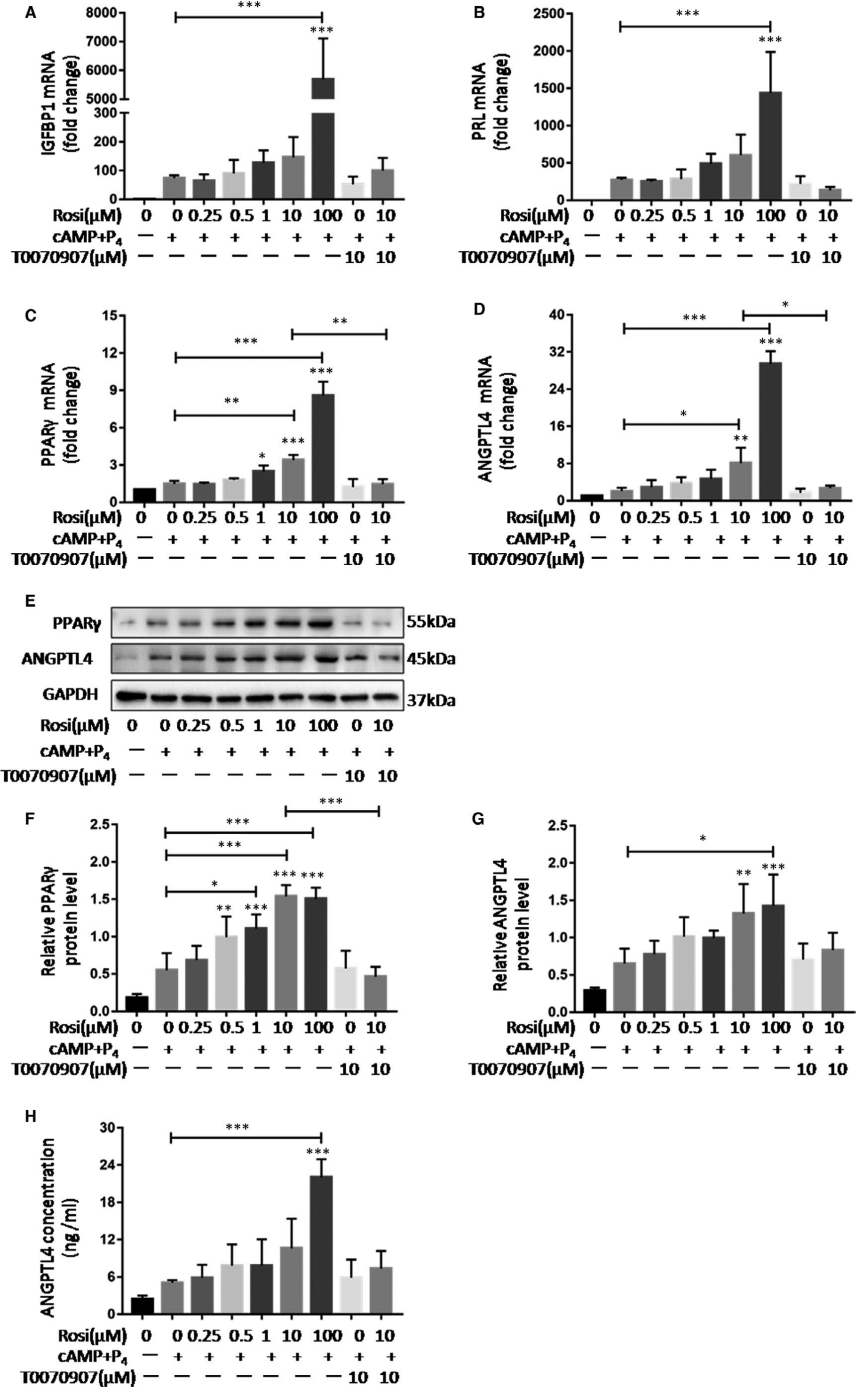
PPARγ mediates rosiglitazone‐induced up‐regulation of ANGPTL4 in hESCs. Decidual cells were treated with rosiglitazone (0, 0.25, 0.5, 1, 10 and 100 μM) for 24 h with pre‐treatment of T0070907 (10 μM) or not. Effect of rosiglitazone on mRNA levels of (A) IGFBP1, (B) PRL, (C) PPARγ and (D) ANGPTL4 in decidual cells. (E‐G) Effect of rosiglitazone on protein abundance of PPARγ and ANGPTL4 in decidual cells. (H) Concentrations of ANGPTL4 in culture medium of decidual cells. The data are shown as the mean ± SD values of at least three independent experiments performed. *P < .05, **P < .01 and ***P < .001 compared with corresponding control

To analyse the role of the PPARγ agonist rosiglitazone in the expression and secretion of ANGPTL4, decidual hESCs were treated with rosiglitazone at various concentrations (0, 0.25, 0.5, 1, 10 and 100 μM) after in vitro decidualization. The concentrations of rosiglitazone were based on previous reports and pilot works in our laboratory (data not shown), and we used 10 μM as the concentration of rosiglitazone treatment as a mode of activation of PPARγ in the current study accordingly.^18^ Significant increases of IGFBP1 and PRL (Figure 3A, B) were detected after rosiglitazone treatment, indicating it has a positive effect on decidualization. In particular, rosiglitazone not only increased both mRNA and protein levels of PPARγ in a concentration‐dependent manner, but also induced ANGPTL4mRNA and protein expression in a similar pattern (Figure 3C‐H). In addition, to further confirm the role of PPARγ in rosiglitazone‐induced expression of ANGPTL4, hESCs were pre‐treated with 10μM PPARγ antagonist T0070907. T0070907 acts as a selective antagonist of PPARγ and inhibits the transcriptional activation of PPARγ in the presence of rosiglitazone. The results showed that blocking the function of PPARγ diminished the elevated levels of ANGPTL4 induced by rosiglitazone (Figure 3C‐H).

**Figure 4:**
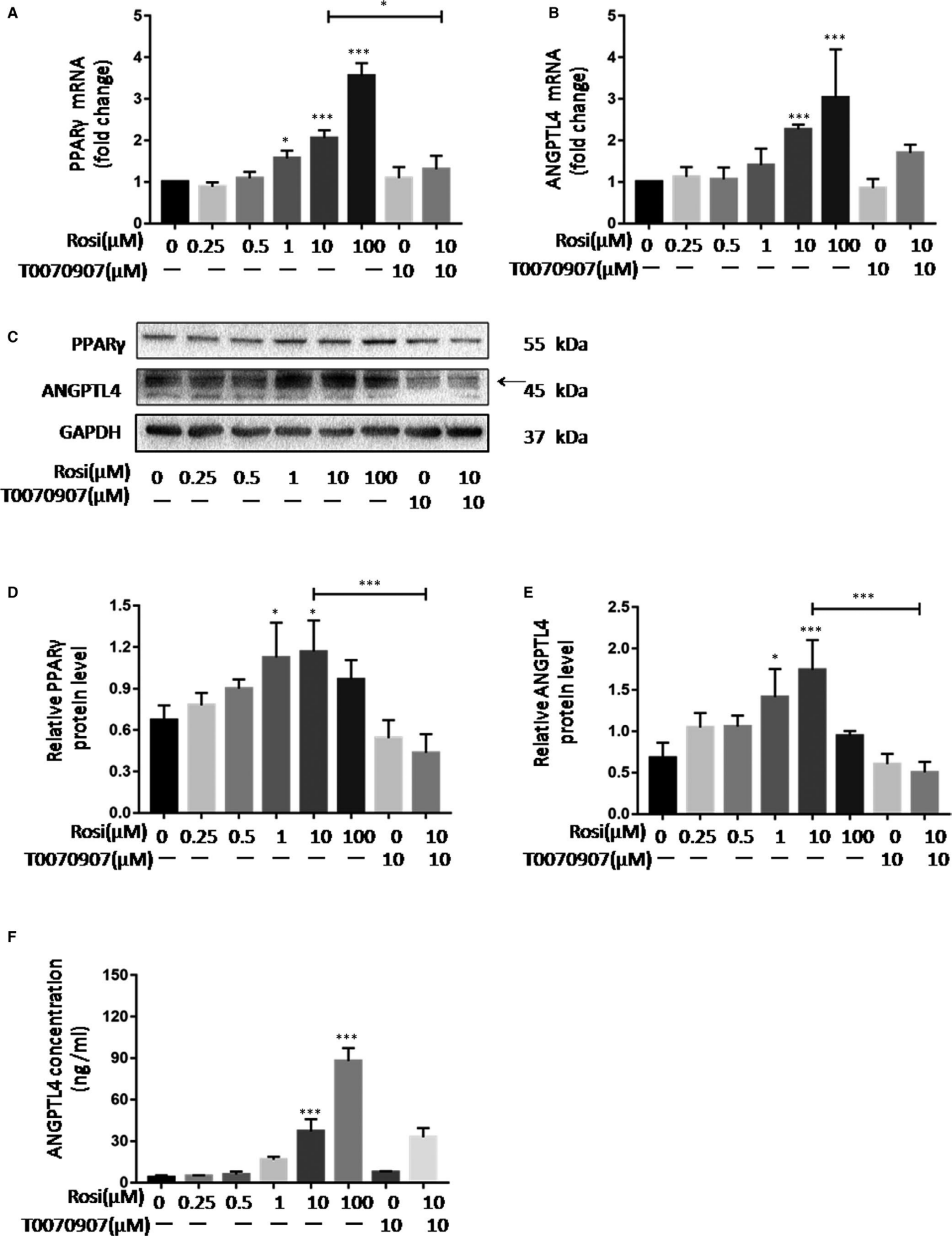
PPARγ mediates rosiglitazone‐induced up‐regulation of ANGPTL4 in HUVECs. HUVECs were treated with rosiglitazone (0, 0.25, 0.5, 1, 10 and100 μM) for 24 h with pre‐treatment of T0070907 (10 μM) or not. (A and B) Effects of rosiglitazone on mRNA levels of PPARγ and ANGPTL4 in HUVECs. (C‐E) Typical bands and statistical results of protein abundance of PPARγ and ANGPTL4 in HUVECs after rosiglitazone treatment. Arrowhead denotes the specific band of ANGPTL4 in HUVECs. (F) Concentrations of ANGPTL4 in culture medium of HUVECs after rosiglitazone treatment. The data are shown as the mean ± SD values of at least three independent experiments performed. *P < .05 and **P < .01 compared with corresponding control

Similar results were observed in HUVECs, rosiglitazone increased both mRNA and protein levels of ANGPTL4 and PPARγ, while the effect of rosiglitazone was partly counteracted by the PPARγ antagonist (Figure 4A‐F). These data indicate that PPARγ mediates rosiglitazone‐induced expression of ANGPTL4.

### 3.5 ANGPTL4 mediates rosiglitazone‐induced HUVECs proliferation

**Figure 5:**
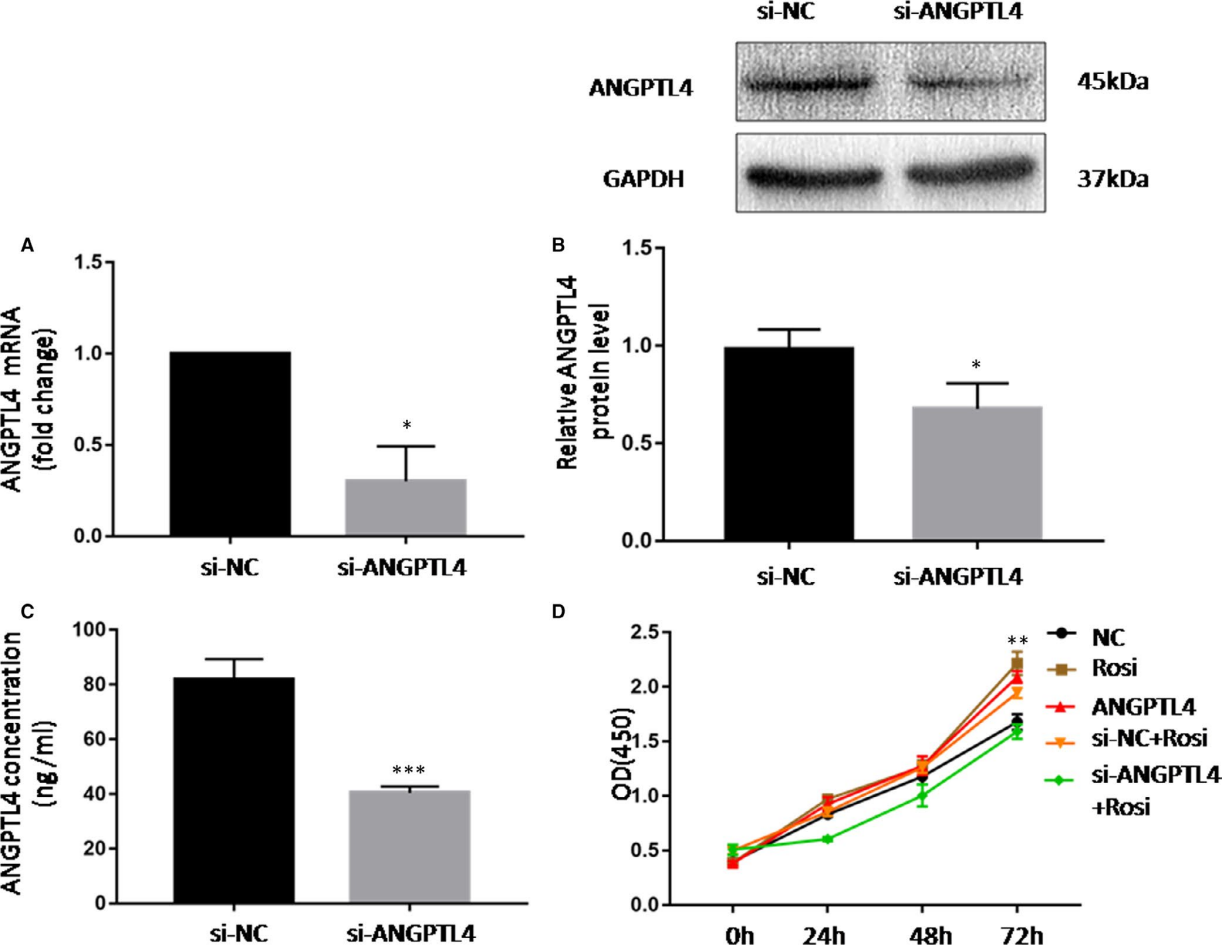
ANGPTL4 mediates rosiglitazone‐induced HUVECs proliferation. (A‐C) mRNA levels, protein levels and concentrations of ANGPTL4 in culture medium after siRNA‐mediated knockdown of ANGPTL4 in HUVECs. (D) Cell counting Kit‐8 assay was performed to examine cell proliferation at the indicated time points on HUVECs after siRNA‐mediated knockdown of ANGPTL4 and followed by treatment of rosiglitazone (10 μM; 24h) or rhANGPTL4 (100 nM; 24h). NC, negative controls. The data are shown as the mean ± SD values of three independent experiments performed, and there are 6 wells for each group in CCK‐8 assay. *P < .05 and **P < .01 compared with corresponding control

HUVECs were transfected with small interfering RNA for 48 h, the abundance of ANGPTL4 was significantly reduced regardless of mRNA levels, protein levels or concentrations in cell culture medium (Figure 5A‐C). The effect of ANGPTL4 on HUVECs proliferation was detected by CCK‐8 assay. Both rosiglitazone and rhANGPTL4 significantly promoted cell proliferation, while the effect of rosiglitazone was partially counteracted by the siRNA‐mediated knockdown of ANGPTL4 (Figure 5D). Taken together, these results demonstrate that ANGPTL4 plays an important role in HUVEC proliferation induced by the PPARγ agonist.

### 3.6 ANGPTL4 is important for rosiglitazone‐induced HUVECs migration

**Figure 6:**
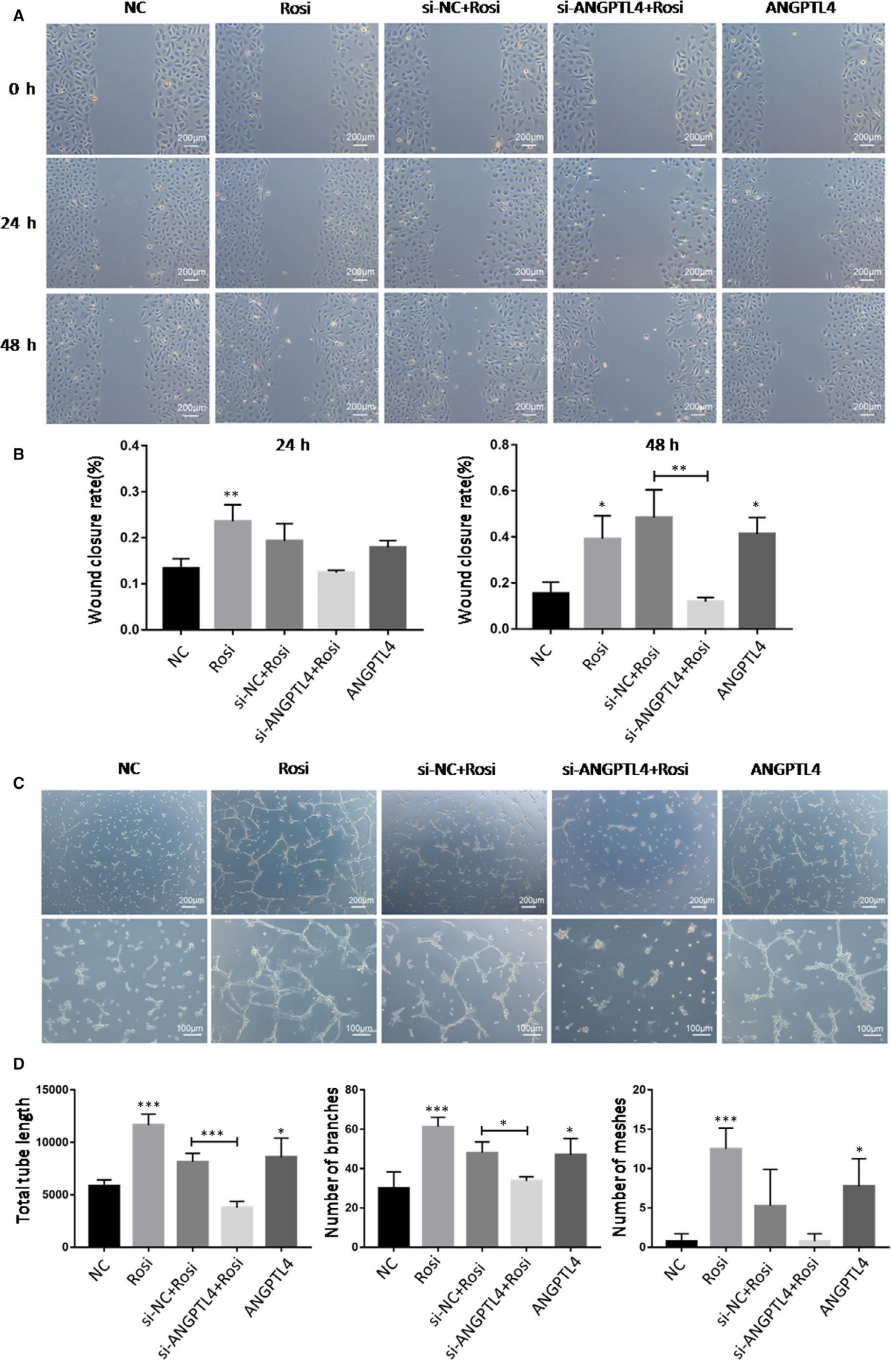
Effect of Rosiglitazone and ANGPTL4 on migration and angiogenesis in HUVECs. Wound‐healing assays were performed to assess the migratory abilities in HUVECs after treatments described as in CCK‐8 assay. Tube formation assay was performed to observe angiogenesis in HUVECs after treatments. (A) Images were taken after cell scratching at 0 h, 24 h and 48 h respectively. (B) Wound closure rates were compared at 24 h and 48 h. (C) Images were taken 24 h after cell plating respectively. (D) Total tube length, number of branches and number of meshes were analysed. NC, negative controls. Scale bar = 100 μm or 200 μm. The data are shown as the mean ± SD values of three independent experiments performed. *P < .05, **P < .01 and ***P < .001 compared with corresponding control

Wound‐healing assays were conducted in HUVECs to explore the role of ANGPTL4 and rosiglitazone in cell migration. Cell migration was compared at 24 and 48 h. Rosiglitazone and rhANGPTL4 had similar effects on the migration of HUVECs, while depletion of ANGPTL4 revoked the effect of rosiglitazone (Figure 6A, B). These observations indicated that PPARγ agonist‐induced cell migration is at least partially related to ANGPLT4.

### 3.7 ANGPTL4 is involved in rosiglitazone‐induced angiogenesis

Tube formation assay was performed to investigate the role of rosiglitazone and ANGPTL4 in angiogenesis on HUVECs. Compared with the control, rosiglitazone and rhANGPTL4 significantly increased the total tube length, the number of branches and the number of meshes while the knockdown of ANGPTL4 partially reversed the effect of rosiglitazone on angiogenesis (Figure 6C, D).

## 4 DISCUSSION

Recurrent implantation failure is generally considered to be closely related to embryo quality and abnormal endometrial receptivity.^19^ The UAI is closely related to the blood supply of endometrium and is a useful tool for identifying impaired uterine circulation, which plays an important role in the establishment of endometrial receptivity.^20^, ^21^ In addition, previous studies have reported uterine perfusion could elevate UAI and impair uterine receptivity, leading to recurrent pregnancy loss (RPL).^20^ Uterine artery blood flow increases progressively during the luteal phase, with the highest flow encountered in the period that temporally coincides with the implantation window.^22^ Accordingly, we conducted uterine arterial blood flow measuring on women undergoing IVF and noted that the UAI of some RIF patients was relatively elevated. Notably, previous studies have found that the uterine artery PI and subendometrial blood flow RI were higher in RIF patients than controls, meanwhile the waveform pattern of uterine artery altered in patients reporting RIF.^23^ However, in the current study, the recruited women who conceived in the control group may show some minor differences in UAI compared with fertile women due to the application of hormones and other drugs during IVF treatment. We observed a significant increase in the uterine artery RI and S/D value in RIF patients compared to the control group, and as a result, we attached more attention on the value of S/D and defined that the values of S/D over 6 on both sides as elevated UAI.

During the process of decidualization, decidual cells secrete various cytokines and growth factors, such as PRL and IGFBP1, defined as markers of decidual cell differentiation, which can regulate trophoblast invasion and spiral arterial remodelling, support embryo development, and provide immune protection for both mother and foetus.^24^, ^25^ our study found that compared with the control group, RIF women with elevated UAI had significantly lower IGFBP1 and PRL mRNA levels in peri‐implantation endometrial tissues, suggesting impaired decidualization in RIF patients. Insufficient endometrial angiogenesis is associated with increased resistance to endometrial blood flow from uterine artery. Previous study revealed a strong link between endometrial vascularity and pregnancy rate, and inadequate endometrial vascularization was associated with pregnancy loss.^26^ Thus, the synchronicity of decidualization and vascularization process is essential for the establishment of endometrial receptivity.

ANGPTL4 is located on chromosome 19p13.3 and consisted of seven exons which encode a 406‐amino‐acid secretory glycoprotein with a molecular mass of 45‐65 kDa.^13^ Many studies have illustrated that ANGPTL4 is a multifunctional factor involved in lipid metabolism, wound healing and angiogenesis.^27^, ^28^ During the secretory period of endometrium, endometrial arched arteries branches form terminal arterioles, spiral arteries bend and expand and vascular smooth muscle cells become distorted to form a low‐resistance vascular network.^11^ Interestingly, previous studies have found that ANGPTL4 is reduced in placental tissues of patients with preeclampsia, suggesting that ANGPTL4 plays an important protective role during embryo development.^29^ In this study, our analysis of peri‐implantation endometrial tissues and serum of RIF women with elevated UAI showed that ANGPTL4 mRNA and protein levels were significantly reduced compared to controls. At the same time, ANGPTL4 increased significantly upon in vitro decidualization on hESCs. These results indicate that ANGPTL4 plays an important role in endometrial angiogenesis and decidualization during WOI.

A number of factors including fasting, hypoxia, glucocorticoid, free fatty acids, PPAR agonists, transforming growth factor‐β (TGF‐β) and hypoxia inducible factor‐1α (HIF‐1α) have been shown to regulate ANGPTL4 transcription.^12^, ^13^, ^30^ During in vitro decidualization experiments of mouse endometrial stromal cells, ANGPTL4 expression increased and PPAR agonist (cPGI2) up‐regulated ANGPTL4 expression.^31^ PPARγ is a ligand‐activated transcriptional factor that is highly expressed in adipocytes and acts as a primary regulator of adipogenesis.^32^ Previous research has found that PPARγ plays an important regulatory role in complex trophoblast lineage differentiation and normal vascular function, which is required for normal placental development.^33^ In human endometrium, PPARγ is mainly located in endometrial stromal cells.^34^ ANGPTL4 is reported to be a target gene of PPARγ, which acts as an activating ligand of PPARγ and binds to PPAR response regulatory element (PPRE) to regulate downstream ANGPTL4 expression.^29^, ^35^ However, the role of PPARγ and ANGPTL4 during decidualization and their correlation in endometrium have not been reported. Our study showed that compared with the control group, RIF women with elevated UAI had significantly lower mRNA and protein levels of ANGPTL4 in peri‐implantation endometrial tissues and serum, and the expression of ANGPTL4 was positively correlated to the expression of PPARγ, suggesting that PPARγ may be an upstream regulator of ANGPTL4 in endometrium.

Rosiglitazone, an effective thiazolidinedione insulin sensitizer, is a potent and selective PPARγ agonist. Many studies have reported that rosiglitazone can increase PPARγ and ANGPTL4 expression in isolated thoracic aorta, epididymal fat pad explants, trophoblast cells and placental explants.^29^, ^36^ However, it is unclear whether the expression of ANGPTL4 is regulated by PPARγ agonists during decidualization. Our study illustrated that after in vitro decidualization, the PPARγ agonist rosiglitazone significantly up‐regulated the expression and secretion of ANGPTL4 in a dose‐dependent manner in hESCs. We also found that rosiglitazone induces PPARγ in the same pattern, which is essential for rosiglitazone‐induced expression of ANGPTL4. When hESCs and HUVECs were treated with a selective PPARγ inhibitor (T0070907), the regulatory effect of rosiglitazone was partially blocked.

Angiogenesis is a process of forming new blood vessels from existing blood vessels through activation, proliferation, elongation or sprouting of endothelial cells. A number of cytokines have been identified as important regulators of angiogenesis, including vascular endothelial growth factor‐A (VEGF‐A), placental growth factor (PIGF), proteases and the angiopoietin family. It has been shown that blockade of VEGFR‐1 disrupts decidual angiogenesis during WOI,^37^ while COX2‐derived prostaglandins target the VEGF system, which is important for uterine angiogenesis during implantation and decidualization.^38^ In addition, endometrial angiogenesis during pregnancy facilitates embryo implantation and improves pregnancy success rate.^23^ In the current study, to further explore the role of ANGPTL4 in angiogenesis, cell proliferation assay, wound‐healing assays and tube formation assay were performed on HUVECs after siRNA‐mediated knockdown of ANGPTL4. The results showed that ANGPTL4 can enhance the ability of cell proliferation, migration and angiogenesis on HUVECs.

In summary, we found that ANGPTL4 abundance in endometrial tissues and serum were reduced in RIF women with elevated UAI, which was significantly correlated with PPARγ expression. PPARγ and ANGPTL4 were up‐regulated in hESCs after decidualization in vitro. Rosiglitazone induced the expression of ANGPTL4 upon decidualization in hESCs and HUVECs via PPARγ. ANGPTL4 promoted the proliferation, migration and angiogenesis of HUVECs in vitro. These findings demonstrated that ANGPTL4 is a vital product of decidualization and participates in decidual angiogenesis. However, the causal relationship between ANGPTL4 and decidualization is not clear, and the specific cellular mechanism how ANGPTL4 derived from decidual cells interact with vascular endothelial cells requires further study. Besides, since the behaviour of immortalized cell lines could not be always replicated by primary cultures from patients, primary ESCs isolated from endometrial tissues of fertile women and RIF patients is required to confirm the effect of rosiglitazone and PPAR inhibitor on ANGPTL4 expression. Moreover, ongoing research on endometrial angiogenesis needs to be further verified in small blood vessels or micro vascular endothelial cells derived specifically from endometrial tissues. Nevertheless, our research has identified the possible cause of RIF in patients presenting elevated UAI and provided a new perspective and potential treatment target.

## CONFLICT OF INTEREST

None declared.

## AUTHOR CONTRIBUTION

**Mingyang Li:** Data curation (equal); Formal analysis (equal); Investigation (equal); Methodology (equal); Project administration (equal); Writing‐original draft (equal). **Jingwen Hu:** Data curation (equal); Formal analysis (equal); Investigation (equal); Methodology (equal); Project administration (equal); Writing‐original draft (equal); Writing‐review & editing (equal). **Lihua Yao:** Conceptualization (equal); Resources (equal). **Minzhi Gao:** Conceptualization (lead); Data curation (lead); Funding acquisition (lead); Resources (lead); Writing‐original draft (lead); Writing‐review & editing (lead).

## Data Availability

Data are available on request from the authors.
